# Effects of Ultrasonication in Water and Isopropyl Alcohol on High-Crystalline Cellulose: A Fourier Transform Infrared Spectrometry and X-ray Diffraction Investigation

**DOI:** 10.3390/polym16162363

**Published:** 2024-08-21

**Authors:** Răzvan Rotaru, Maria E. Fortună, Elena Ungureanu, Carmen O. Brezuleanu

**Affiliations:** 1 “Petru Poni” Institute of Macromolecular Chemistry, Department of Inorganic Polymers, 41A Grigore Ghica Voda Alley, 700487 Iasi, Romania; rotaru.razvan@icmpp.ro; 2 “Ion Ionescu de la Brad” Iasi University of Life Sciences, 3 Mihail Sadoveanu Alley, 700490 Iasi, Romania; olgutabrezuleanu@iuls.ro

**Keywords:** alcohol, cellulose, ultrasonication, water

## Abstract

This paper investigates the effects of ultrasonication on cellulose microparticles in different conditions. FTIR (Fourier transformed infrared spectrometry) and XRD (X-ray diffraction) analyses were used to compare the changes in the cellulose microstructure caused by the following various ultrasonic treatment conditions: time, amplitude of generated ultrasound waves, output power converted into ultrasound, the liquid medium (water and isopropyl alcohol) used for ultrasonication, and the shape of the vessel used for sonication. The cumulative results lead to an increase in the crystalline region directly proportional to the condition of sonication. Also, the total crystallinity index varied from 1.39 (pristine cellulose) to 1.94 for sonication in alcohol to 0.56 for sonication in water. The crystallinity index varied from 67% (cellulose) to 77% for the sample with 15 min of sonication in isopropyl alcohol and 50.4% for the sample with 15 min of sonication in water.

## 1. Introduction

Due to its abundance and renewable nature, cellulose is perhaps the most significant biopolymer on earth and has been a part of human life since the dawn of time (such as paper woven from cotton). Over the last 150 years, there have been many uses for cellulose, its derivatives, and its composites, including the manufacturing of food and paper [1,2], bio- and pharmaceutical materials, magneto-optical and recyclable catalyst applications, the electrotechnical industry, etc. [3,4]. In its natural form, this polymer is made by a linear chain of anhydroglucose molecules united by a β-1, 4-glycosidic bond [5]. Due to the hydrogen bonds and van der Waals forces holding these chains together, cellulose is insoluble in most common solvents [6]. In order to extract cellulose from biomass, obtain composites or derivatives, or modify structure and morphology, cellulose is treated in different ways. The nanoparticles of crystalline cellulose are obtained from the acid hydrolysis of cellulose fibers [7]. Ionic liquid (1-butyl-3-methylimidazolium chloride, 1-ethyl-3-methylimidazolium tetrachloroaluminate) is used as a pretreatment for cellulosic materials [6,8], and as a result, there appears to be variation in the crystallinity index. A new method for generating cellulose composites is presented using the ultrasonic technique. W. Chen used hard ultrasonication (20–25 kHz ultrasound frequency, 1000 watts of output power, and 30 min of sonic treatment) to obtain cellulose nanofibers from plant cellulose fibers with a noticeably increased crystallinity (in the case of wood used as a source of cellulose, there was an increase from 56% in raw materials to 71–73.2% on ultrasonic-treated cellulose) [9]. X.F. Li and his colleagues [10] created spherical nano-crystal cellulose from cellulose I and II using a novel technique that involved acid hydrolysis and ultrasonication (20 kHz, 400 W output power, and more than 8 h of ultrasound irradiation). The time of the ultrasonic treatment was correlated with the shape and degree of the scatter of particles.

Using high-intensity ultrasonication (20 kHz, 1500 W, and varying times: 5, 10, and 15 min), rod-shaped nanocrystalline cellulose was formed from microcrystalline cellulose. In this case, the time of ultrasonication directly affected the length of rod-shaped cellulose [11]. A.N. Frone and collaborators managed to prepare cellulose nanofibers from microcrystalline cellulose and then obtained composites with nanofibers and poly (vinyl alcohol) via the ultrasonication technique (20 kHz, 500 W, 10–20 min) [12]. Using a chemical procedure and high-intensity ultrasonication (20–25 kHz frequency, 400–1200 W output power, and 30 min of ultrasonication), W. Chen and associates extracted cellulose nanofibers from poplar trees [13]. They found that the diameter distributions of the nanofibers, which have a crystallinity of roughly 69%, depend on the output power (0.4–1.2 kW) of the ultrasonic treatment. S. Parveen and associates developed cellulose-reinforced cementation composites by homogeneously dispersing microcrystalline cellulose using ultrasonication energy (45 kHz frequency and 80 W power) [14]. Ultrasonication was carried out for different time periods, such as 15, 30, 45, and 60 min, in order to investigate the influence of the time of ultrasonication on dispersion quality. In another study, Girard et al., used the ultrasonication modeling methodology to investigate the ultrasonic (20 kHz frequency) dispersion of cellulose nanocrystals. They used the solution of a coupled problem between the Navier–Stokes equations and a steady-state variant of the wave equation [15]. Using numerical modeling, several key parameters were identified to obtain a well-dispersed CNC suspension, such as the beaker geometry, the probe position, and, more specifically, depth and centering. A.M. Barbosa and associates obtained cellulose nanocrystal membranes as excipients for drug delivery systems from flax fibers via acid hydrolysis assisted by sonochemistry [16]. These nanocrystals of cellulose exhibited a high crystallinity, and the ultrasonic process was conducted at a frequency of 37 kHz for different periods of time: 30, 45, and 60 min. Wong et al. studied the effect of prolonged ultrasonication on the molecular weight and crystallinity index of cellulose (bacterial and plant cellulose) [17]. They found a reduction in molecular mass and crystallinity index. The ultrasound process (37 kHz frequency and 150 W powers) was conducted in a cuprammonium hydroxide solution for 5, 10, 15, 30, 60, and 90 min. In general, it can be said that the synthesis, dispersion, or homogenization of cellulose by ultrasound in certain media can cause a change in crystallinity, shape, or hydrogen bonds. Thus, the reactivity of cellulose can be modified, and this can matter a lot, especially in medical applications [18]. In a previous investigation, we discovered that ultrasonic treatment in water causes cellulose with fiber lengths of 20 microns, whether in composite or alone, to lose part of its crystallinity [4,19]. In a recent investigation (unpublished), we discovered that high-crystalline cellulose with fiber lengths of 1 micron ultrasonicated in different types of alcohol (pristine or in composites with different inorganic particles) presents an increase in crystallinity. The purpose of this investigation is to conduct research on the behavior of micronized cellulose under ultrasonic waves in water and alcohol.

## 2. Materials and Methods

### 2.1. Materials

Microcrystalline cellulose (C, Avicel, Wilmington, Delaware, U.S.CAS 9004-34-6, grade PH-101) was used with density = 1.5 g/cm^3^ (20 °C), bulk density = 70–400 kg/m^3^, isopropyl alcohol (IPA, Sigma-Aldrich, Burlington, MA, USA) with Mw = 60.1 g/mol, and a purity of 99.5%. Milli-Q ultrapure-distilled water (H_2_O, our laboratory) was used without further purification.

Ultrasonic irradiation of cellulose

Each 2 g sample of pristine cellulose (C) was ultrasonicated in 70 mL of liquid (isopropyl alcohol or water) in a 100 mL beaker under various conditions (time: 5, 10, and 15 min of ultrasonication, amplitude of ultrasonic wave: half (50%) or the whole (100%), liquid medium for ultrasonication: isopropyl alcohol or water; and vessel for ultrasonic process: cylindrical Berzelius or spherical-round-bottomed flask) as shown in Table 1. Following decantation (24 h at room temperature), the samples were dried in a vacuum oven at 50 °C overnight. Each sample was collected as a fine white powder.

### 2.2. Methods

The ultrasonication experiments were performed using a Sonic Vibracell ultrasonic generator with a nominal electric power of 750 W and an ultrasound frequency of 20 kHz, provided with a display giving the energy delivered to the end of the probe and a temperature sensor. The samples were dried in a Trade-Raypa vacuum oven at 50 °C.

FTIR spectroscopy was performed using a Bruker Vertex 70 spectrometer on potassium bromide (KBr) pellets with a 2 cm^−1^ resolution. The concentration of the samples was a constant of 2 mg/200 mg of KBr.

To calculate the empirical crystallinity index, or lateral order index (LOI), the following equation was used:

LOI = I_1435_/I_899_ (1)

where I is the intensity of the absorbance peak, and the wavenumbers are 1435 and 899 cm^−1^, respectively.

The total crystallinity index (TCI) was determined using Equation (2):

TCI = I_1366_/I_2914_ (2)

The hydrogen bond intensity (HBI) was established with the following ratio:

HBI = I_3339_/I_1323_ (3)

The energy of the hydrogen bonds (noted E_H_) for several OH stretching bands was calculated using Formula (4):

(4) $$E_{H}=\frac{1}{k}\cdot \frac{\nu o-\nu }{\nu }$$

where k = 1.68 × 10^−2^ kcal^−1^, ν_0_ is the standard frequency corresponding to free OH groups (3600 cm^−1^), and ν is the frequency of the bonded OH groups [20,21].

Pimentel and Sederholm [22] proposed Equation (5), which yields the hydrogen bond distances, R, as follows:

Δ(ν) = 4430(2.84 − R) (5)

where Δ(ν) = ν_0_ − ν, ν_0_ is the monomeric OH stretching frequency, which is taken to be 3600 cm^−1^, and ν is the stretching frequency observed in the infrared spectrum of the sample (deconvolution spectra).

X-ray diffraction (XRD) on a Bruker Advance D8 X-ray diffractometer (λ: 1.5405 Å, with the wavelength of Cu-Kα radiation, 2θ, ranging from 4 to 60°).

The values of the crystallinity index (C_r_I) of cellulose and sonic-treated samples were obtained with the Segal method according to Equation (6) [23]:

(6) $$C_{r}I=100\cdot \frac{I002-Iam}{I002}\%$$

where I_002_ represents the maximum intensity of the 002 lattice diffraction with a peak corresponding to a 2θ angle around 22–24°, and I_am_ is the intensity of diffraction of the non-crystalline (amorphous) material, which is taken at a 2θ angle of about 18°.

The average size of the crystallites, measured in the directions orthogonal to the (101), (10$\bar{1}$), and (002) planes, is calculated using the Scherrer Formula (7):

(7) $$D=\frac{K\cdot \lambda }{\beta \cdot cosƟ}$$

where D is the average crystallite size, K is the Scherrer constant (0.89), λ is the wavelength of the incident X-ray (Kα1 = 1.5406 Å, Kα2, 1.5443 Å. and Kβ, 1.3923 Å), β represents the full width at half maximum of the reflection in the radial direction (diffraction band), and θ is the angle corresponding to the crystalline peak (Bragg angle) [21,24]. We filtered out the Kβ radiation, leaving a weighted average of 1.5418 Å with a Ni filter. Lattice parameters and lattice volume were calculated with the decomposition method using Origin software, 8 Pro.

## 3. Results and Discussion

### 3.1. The Chemical Structure of the Irradiated Samples Established by FTIR Analysis

**Figure 1:**
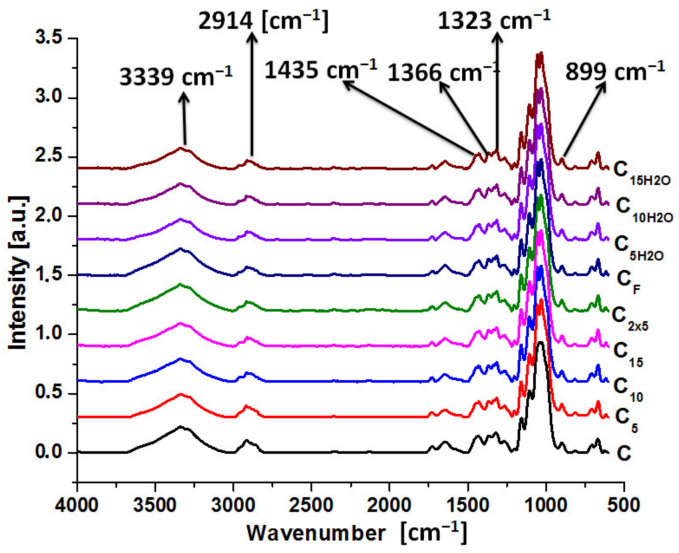
Infrared spectra for pristine cellulose and ultrasonicated samples.

Infrared spectroscopy was used to examine the structure of both pure cellulose and ultrasonic-irradiated samples. Figure 1 presents the infrared spectra for pristine cellulose (C) and ultrasonic-irradiated samples (C_5_, C_10_, C_15_, C_2x5_, C_F_, C_5H2O_, C_10H2O,_ and C_15H2O_).

The type of vibration and wave number are given in Table 2. For sonicated samples, some peaks were practically unmodified, but there were also noticeable changes.

Except for the hydroxyl groups, the largest displacements of the wave number were found at ν_CH_ vibrations (2914 cm^−1^ for pristine cellulose) from 48 to 65 cm^−1^. The smallest displacements were observed for ν_CO-_ and δ_CH2_-type vibrations (1055, 1030, and 899 cm^−1^) from 4 to 8 cm^−1^.

Some of the characteristic spectral bands were quite sensitive to the crystalline structure in cellulose materials. Thus, the bands located at 2914, 1435, 1366 cm^−1^, and 899 cm^−1^ corresponded to crystalline and amorphous domains, respectively [4]. So, TCI, LOI, and HBI were calculated with Formulas (1)–(3), and the results of these parameters are shown in Table 3. Taking into account chain mobility, bond distance, and the amount of bounded water, the TCI is proportional to the crystallinity of cellulose, the LOI is the overall degree of order in cellulose, and the hydrogen bond intensity HBI refers to the crystal system and the degree of intermolecular regularity.

For ultrasonication in isopropyl alcohol, the lateral order index (LOI) suffers a small decrease, indicating that ultrasonic irradiation directly affects the overall degree of order in cellulose, but the total crystallinity index (TCI) is increased, and so the crystallinity degree of cellulose is bigger. The ultrasonication time is directly proportional to this crystallinity increase (TCI for 5 min is 1.45, 10 min is −1.72, and 15 min is −1.89).

The intensity of ultrasonic irradiation is directly proportional to the amplitude of the vibration of the sonic source. Therefore, an increment in the amplitude leads to an increase in the intensity of vibration and an increase in the sonochemical effects (for the main microcavitation effect, bubble formation is observed, followed by their growth and, finally, implosion, which can cause temperatures of approximately 5000 °C in gas phases or more than 2000 °C in liquid phases and pressures higher than 500 atmospheres). Bubble collapse during cavitation serves as a very effective means of concentrating the power energy of the sound: the compression of gas always generates heat. When the compression of bubbles occurs during cavitation, heating is much more rapid than usual thermal transport, creating a short-lived localized hot spot. Ultrasonic irradiation is different from traditional energy sources (e.q., heat, light, or ionizing radiation) in duration, pressure, and energy per molecule. The immense local temperatures and pressures, together with the extraordinary heating and cooling rates generated by cavitation bubble collapse, mean that ultrasound provides a very unique mechanism for generating high-energy chemistry [25]. By doubling the amplitude of the wave (100%) for 5 min, TCI is almost equivalent to 10 min of ultrasonication at a 50% amplitude (TCI C_2x5_ = 1.75~TCI C_10_ = 1.72 compared with TCI C_5_ = 1.45). The shapes of the vessels also affect the TCI index (TCI-C_F_ = 1.93 compared with TCI-C_5_ = 1.45). It is already known that a round-bottomed flask minimizes the “dead zone” for ultrasound wave action better than a Berzelius beaker [26].

Isopropyl alcohol is commonly employed in ultrasonic synthesis for the deagglomeration of powders [27]. Water, like ultrasonication liquid medium, has a different effect than alcohol (TCI-C_5_H_2_O = 1.94, the highest value) [28].

However, when the ultrasound time is increased to 10 and 15 min, the TCI index significantly decreases to 0.76 for C_10H2O_ and 0.56 for C_15H2O_ [4]. Another aspect is represented by hydrogen bonding. This is considered to be responsible for various properties of cellulose. Thus, the closer or more distant the cellulose chains are, the larger or smaller the interactions between them, resulting in more and stronger hydrogen bonds or, conversely, weaker connections.

The broadband in the FTIR spectra between 3000 and 3700 cm^−1^ corresponds to the hydroxyl stretching vibration and offers information about hydrogen bonding (intra and intermolecular).

**Figure 2:**
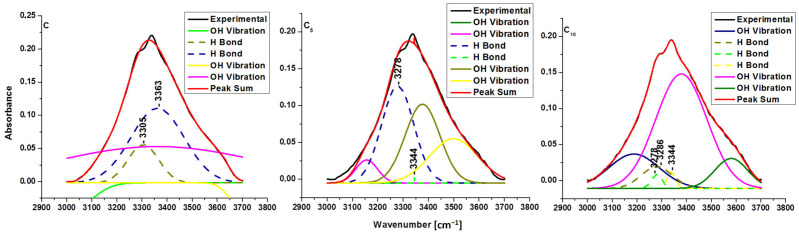
Deconvoluted FTIR spectra (range 300–3700 cm^−1^) for C, C_5_, and C_10_ samples.

**Figure 3:**
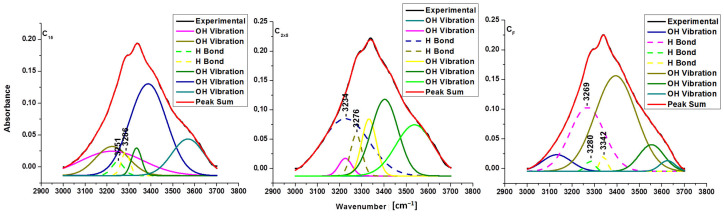
Deconvoluted FTIR spectra for C_15_, C_2x5_, and C_F_ samples.

**Figure 4:**
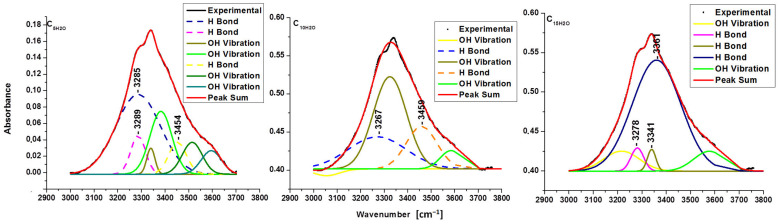
Deconvoluted FTIR spectra for C_5H2O_, C_10H2O_, and C_15H2O_ samples ultrasonicated in water.

The assignments of intramolecular hydrogen bonds are shown for O(2)H-O(6) in the cellulose crystalline structure at 3410–3460 cm^−1^, for intramolecular O(3)H-O(5) at 3340–3375 cm^−1^ and for the intermolecular hydrogen bonding of O(6)H-O(3) in the region 3230–3310 cm^−1^ [14,27]. Since the absorbance peaks of pristine celluloses and sonicated samples in this range (3000–3700 cm^−1^) were overlapped, the resolution of the spectra was improved by their deconvoluted graphics from background scattering using a Gaussian function curve—multiple peak fit analysis using Origin 8.5 Pro software. The energy of hydrogen bonds and hydrogen bond distances for pristine cellulose and sonicated samples are presented in Table 4, and the deconvoluted spectra in 3000–3700 cm^−1^ regions are presented in Figure 2, Figure 3 and Figure 4.

In black, the experimental curve obtained from the infrared spectrometer is shown, and in red, the theoretical curve obtained from the sum of all deconvoluted curves is shown. Ideally, black and red curves need to overlap. The small differences represented hydroxyl groups from the atmosphere (moisture) or small errors from the infrared sensor.

It is observed that the intermolecular hydrogen bond O(6)H-O(3) changes energy (an increase with sonication) and distances (a decrease with sonication). Ultrasonication reduces hydrogen bond distances in pure cellulose for 5, 10 min, and for 15 min, indicating a minor decline in the linear chain of anhydroglucose molecules. The high energy and the small distances were obtained for C_2x5_ when the amplitude of ultrasound was double. Table 4 shows how the shape of the reaction vessel (flask/Berzelius) and the ultrasonication time had a direct influence on the energy of hydrogen bonding.

The reduced distances for the intermolecular hydrogen bond O(6)H-O(3) in sonic-irradiated samples increased crystallization by facilitating the ordering of the linear chain of glucose residue.

### 3.2. Crystalline Structure of the Ultrasonicated Sample: X-ray Diffraction Analysis

**Figure 5:**
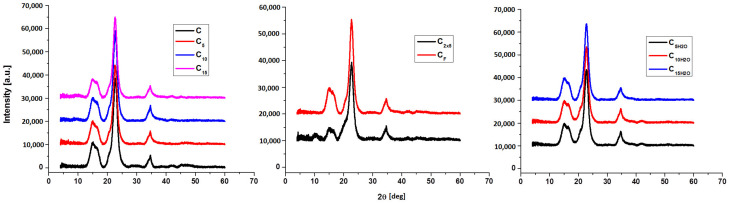
XRD pattern for pristine cellulose powder and ultrasonicated samples.

X-ray diffraction (XRD) analysis was used to accomplish a deeper structural characterization (in order to measure the crystallinity, apparent crystallinity, and size of the crystallites) of the studied cellulose and sonicated samples. Figure 5 depicts the diffractograms for native cellulose and sonicated materials, while Table 5 provides quantitative and qualitative data (space group, crystal system, lattice parameters, lattice volume, and crystallite size).

**Figure 6:**
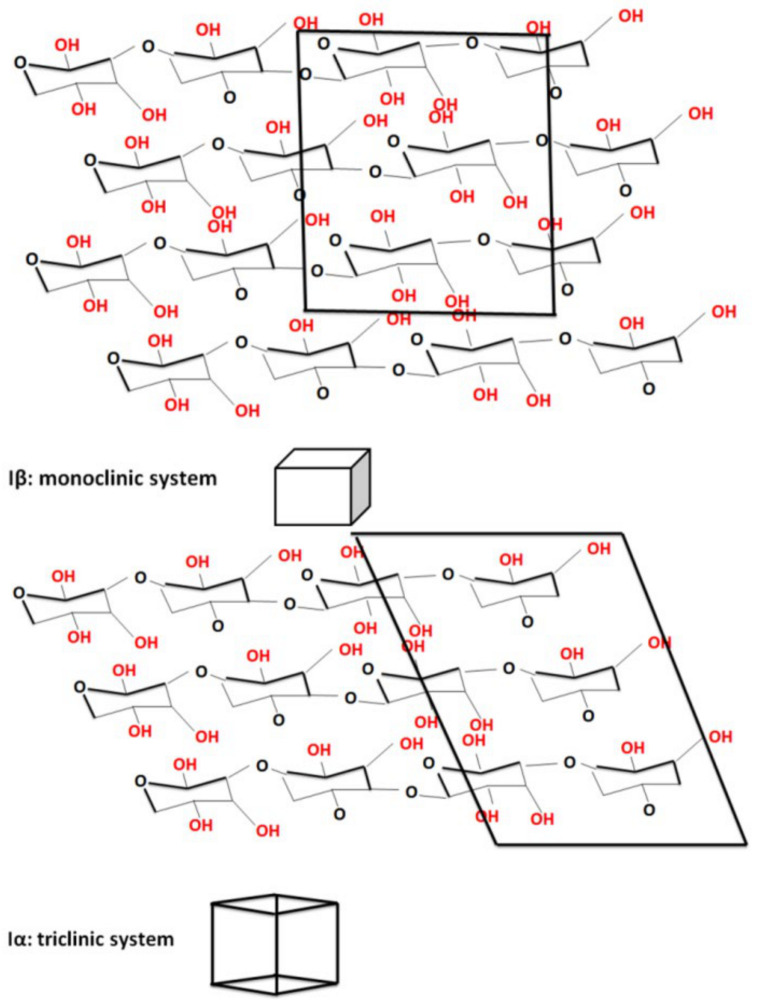
Monoclinic and triclinic cellulose system.

The X-ray diffractograms of pristine celluloses and sonicated samples show the characteristic shapes of the cellulose I crystalline structure corresponding to crystallographic (1–10), (110), and (200) planes at Bragg angles of 14.71°, 16.63°, and 22.55°, respectively. Figure 6 shows that ultrasonication in isopropyl alcohol changes the space group (from P1121 to 1: P1) and the crystalline structure (from monoclinic to triclinic), whereas ultrasonication in water does not change these parameters.

Alcohol-treated samples saw a significant decrease in lattice volume (from 647 to 331–342 Å), while Milli-Q Watter treatment resulted in a modest increase to 676–695 Å. In order to correctly calculate the values of the crystallinity index (CrI), the resulting diffraction patterns were deconvoluted from background scattering using a Gaussian function curve-fitting analysis.

**Figure 7:**
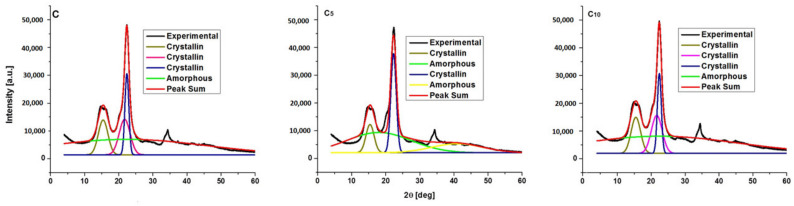
Deconvoluted X-ray diffractograms for C, C_5_, and C_10_.

**Figure 8:**
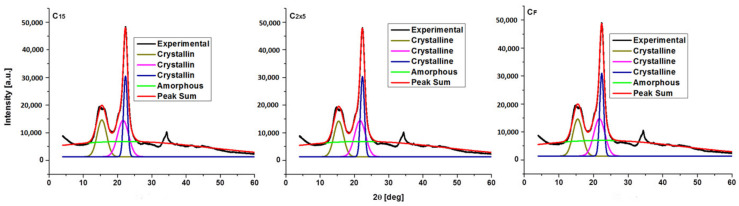
Deconvoluted X-ray diffractograms for C_15_, C_2x5_, and C_F_.

**Figure 9:**
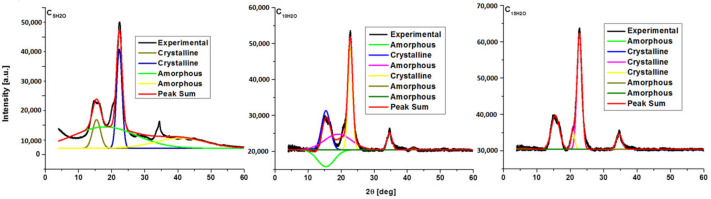
Deconvoluted X-ray diffractograms for C_5H2O_, C_10H2O_, and C_15H2O_.

Deconvoluted diffractograms for native cellulose and sonicated samples are depicted in Figure 7, Figure 8 and Figure 9, and data for the crystallinity index are given in Table 6.

Before deconvolution, the X-ray diffraction pattern exhibits a big peak at 2θ, 14.8° (secondary peak), and a noticeable peak (major peak) at 2θ, 22.6° [29,30]. There are changes after deconvolution, and these are not limited to peak intensity.

For C, C_10_, C_15_, C_2x5_, CF, and C_15H2O,_ three sharp peaks indicate three crystalline regions with three types of crystallites, with 2θ angles of 15.3°, 21.5°, and 22.6° corresponding to the (10), (020), and (200) planes. C_5_, C_5H2O_, and C_10H2O_ samples show two crystalline peaks only at 15.3° and 22.6° (planes (10) and (200)). The ultrasonication of cellulose in alcohol causes an increase in the crystallinity index, from 67 to 74–77%, and a decrease in the lattice volume, while the ultrasonication procedure in Milli-Q water causes a decrease in the crystallinity index (from 67 to 50–66%) and a slight increase in lattice volume.

The crystalline region is affected, probably through the realignment of the cellulose microfibers. According to data from the literature [4], for long fibers (20 μm), fragmentation appears, which leads to a decrease in crystallinity. For short fibers (1–2 μm), this realignment could lead to an increase in crystallinity. This increase in crystallinity is in accord with the Z.Z. Chowdury study (ultrasonication and acid treatment provide nanocrystalline cellulose with lengths of 0.9–0.1 μm and an 88.3 crystallinity index) [3,9]. For comparison, Table 7 gives some results with an increase or decrease in the crystallinity index compared with the lengths of cellulose fibers.

## 4. Conclusions

In this study, microscopic particles of cellulose were subjected to ultrasonication in isopropyl alcohol and Milli-Q water. It was discovered that when ultrasonic irradiation was performed in alcohol, the crystallinity region suffered an increase proportionally with the conditions of the sonic treatment (time, amplitude of the generated ultrasound wave, and the shape of the vessel used for sonication), while when it was made in water, there was degradation in the crystalline region. This mechanism is different in water and alcohol because these two liquids have different properties (specific density, specific weight), and thus, the ultrasonic waves propagate differently.

## Figures and Tables

**Table 1:** Sample notations and the conditions of ultrasonic irradiation.

Sample	Time [min]	Amplitude [%]	Liquid	Vessel
C	-	-	-	-
C_5_	5	50	C_3_H_7_OH	Berzelius
C_10_	10	50	C_3_H_7_OH	Berzelius
C_15_	15	50	C_3_H_7_OH	Berzelius
C_2x5_	5	100	C_3_H_7_OH	Berzelius
C_F_	5	50	C_3_H_7_OH	Round-bottomed flask
C_5H2O_	5	50	H_2_O	Berzelius
C_10H2O_	10	50	H_2_O	Berzelius
C_15H2O_	15	50	H_2_O	Berzelius

**Table 2:** Wavelength (W) shifts in ultrasound samples (ΔW = W_C_ − W_ultrasonicated sample_).

W_C_ [cm^−1^]	Type of Vibration	W_samples_ [cm^−1^]	Sample	ΔW [cm^−1^]
3339	hydroxyl groups	3336	C_5_	3
		3341	C_F_, C_5H2O_	−2
		3352	C_10H2O_	−13
		3435	C_15H2O_	−96
2914	ν_CH_	2918	C_5_	−4
		2916	C_10_	−2
		2962	C_10_	−48
		2897	C_10_	17
		2962	C_15_	−48
		2851	C_15_	63
		2916	C_2x5_	−2
		2955	C_2x5_	−41
		2899	C_2x5_	15
		2962	C_F_	−48
		2901	C_F_	−13
		2962	C_5H2O_	−48
		2897	C_5H2O_	17
		2849	C_5H2O_	65
		2903	C_10H2O_	11
		2959	C_15H2O_	−45
		2905	C_15H2O_	9
1435	δ_CH2_	1429	C_5_, C_10_, C_15_, C_2x5_, C_5H2O_	6
		1427	C_F_	8
		1433	C_10H2O_	2
		1437	C_15H2O_	−2
1366	δ_CH_, ν_COO_	1367	C_5_, C_10_, C_15_, C_2x5_, C_F_, C_5H2O_	−1
		1371	C_10H2O_	−5
		1375	C_15H2O_	−9
1323	CH_2_	1315	C_5_, C_10_, C_15_, C_2x5_, C_F_, C_5H2O_	8
		1319	C_10H2O_, C_15H2O_	4
1157	ν_CO_, δ_OH_	1159	C_5_, C_10_, C_15_, C_2x5_, C_F_, C_5H2O_, C_10H2O_, C_15H2O_	
1055, 1030	ν_CO_	1059, 1032	C_10H2O_	−4, −2
		1059, 1038	C_15H2O_	−4, −8
899	δ_CH2_	895	C_10H2O_, C_15H2O_	4

**Table 3:** Lateral order index (LOI), total crystallinity index (TCI), and hydrogen bond intensity (HBI) of pristine cellulose and sonicated samples.

Sample	LOI	TCI	HBI
C	1.68	1.39	1.29
C_5_	1.22	1.45	1.19
C_10_	1.45	1.72	1.40
C_15_	1.50	1.89	1.39
C_2x5_	1.46	1.75	1.37
C_F_	1.64	1.93	1.36
C_5H2O_	1.48	1.94	1.23
C_10H2O_	2.05	0.76	2.12
C_15H2O_	1.29	0.56	2.92

**Table 4:** Energy bonds and hydrogen bond distances.

Sample	Type of Hydrogen Bond	E_H_ [kJ]	R [Å]
C	intermolecular O(6)H-O(3)	E_3305_: 23.43	R_3305_: 2.773
	intramolecular O(3)H-O(5)	E_3363_: 18.49	R_3363_: 2.786
C_5_	intermolecular O(6)H-O(3)	E_3278_: 25.78	R_3278_: 2.767
	intramolecular O(3)H-O(5)	E_3344_: 20.09	R_3344_: 2.782
C_10_	intermolecular O(6)H-O(3)	E_3278_: 25.78	R_3278_: 2.767
	intermolecular O(6)H-O(3)	E_3286_: 25.08	R_3286_: 2.769
	intramolecular O(3)H-O(5)	E_3344_: 20.09	R_3344_: 2.782
C_15_	intermolecular O(6)H-O(3)	E_3251_: 28.17	R_3251_: 2.761
	intermolecular O(6)H-O(3)	E_3286_: 25.08	R_3286_: 2.769
C_2x5_	intermolecular O(6)H-O(3)	E_3234_: 29.70	R_3234_: 2.757
	intermolecular O(6)H-O(3)	E_3276_: 25.96	R_3276_: 2.766
C_F_	intermolecular O(6)H-O(3)	E_3269_: 26.57	R_3269_: 2.765
	intermolecular O(6)H-O(3)	E_3280_: 25.60	R_3280_: 2.767
	intramolecular O(3)H-O(5)	E_3342_: 20.26	R_3342_: 2.781
C_5H2O_	intermolecular O(6)H-O(3)	E_3285_: 25.17	R_3285_: 2.768
	intermolecular O(6)H-O(3)	E_3289_: 24.82	R_3289_: 2.769
	intramolecular O(2)H-O(6)	E_3454_: 11.09	R_3454_: 2.807
C_10H2O_	intermolecular O(6)H-O(3)	E_3267_: 26.75	R_3267_: 2.764
	intramolecular O(2)H-O(6)	E_3459_: 10.70	R_3459_: 2.808
C_15H2O_	intermolecular O(6)H-O(3)	E_3278_: 25.78	R_3278_: 2.798
	intramolecular O(3)H-O(5)	E_3341_: 20.34	R_3341_: 2.781
	intramolecular O(3)H-O(5)	E_3361_: 18.66	R_3361_: 2.786

**Table 5:** Quantitative and qualitative data.

Sample	JCPDS Card No	Space Group	Crystal System	Lattice Parameters [Å]			Lattice Volume [Å]	Crystallite Size [Å]
				a	b	c		
**C**	4114994	4:P1121	Monoclinic (C-unique)	7.82	8.03	10.35	647.9	41
**C_5_**	4114383	1:P1	Triclinic	10.45	6.56	6.03	331.6	49
**C_10_**	4114383	1:P1	Triclinic	10.43	6.46	5.93	332.2	46
**C_15_**	4114383	1:P1	Triclinic	10.26	6.49	5.85	324.6	56
**C_2x5_**	4114383	1:P1	Triclinic	10.40	6.61	5.91	317.4	27
**C_F_**	4114383	1:P1	Triclinic	10.32	6.53	5.97	342.1	63
**C_5H2O_**	4114994	4:P1121	Monoclinic (C-unique)	8.02	8.37	10.40	695.3	39
**C_10H2O_**	4114994	4:P1121	Monoclinic (C-unique)	7.96	8.25	10.38	680.6	54
**C_15H2O_**	4114994	4:P1121	Monoclinic (C-unique)	7.98	8.15	10.43	676.3	36

**Table 6:** Crystallinity index for cellulose and sonicated samples.

Samples	C_r_I [%]
C	67.6
C_5_	74.2
C_10_	74.6
C_15_	77.8
C_2x5_	77.4
C_F_	77.6
C_5H2O_	66.9
C_10H2O_	51.6
C_15H2O_	50.4

**Table 7:** Crystallinity index of ultrasonicated cellulose in different conditions.

Sample	Lenghts of Fiber [μm]	Treatment	Time of Treatment [min]	CI [%] before Treatment	CI [%] after Treatment	References
cellulose nano-crystals	0.105	ultrasound assisted acid hydrolysis	35	55.3–63.8-72.4	88.3	[3]
cellulose microfibers	1	ultrasonication in isopropyl alchool	5–15	67.6	72.9–77.6	[this work]
cellulose microfibers	1	ultrasonication in Milli-Q water	5–15	67.6	50–4–66.9	[this work]
cellulose microfibers	20	ultrasonication in Milli-Q water	15	42.5	42.4	[4]
cellulose microfibers with anorganic nanoparticle	20	ultrasonication in Milli-Q water	15	42.5	9.6	[4]
cellulose microfibers	20	ultrasonication in sodium hydroxide solution (1–10%)	240	77	73.8	[29]
cellulose microfibers	50	ultrasonication in sodium hydroxide solution	240	79.8	68.2	[29]
cellulose microfibers	100	ultrasonication in sodium hydroxide solution	240	78.8	67.7	[29]
cellulose microfibers	180	ultrasonication in sodium hydroxide solution	240	76.5	71.7	[29]
cellulose microfibers	180	ultrasonication in sodium hydroxide solution	120	76.5	71.65	[29]

## Data Availability

Data are contained within the article.
